# Targeting the Hexosamine Biosynthetic Pathway Prevents *Plasmodium* Developmental Cycle and Disease Pathology in Vertebrate Host

**DOI:** 10.3389/fmicb.2019.00305

**Published:** 2019-02-28

**Authors:** Pollyanna Stephanie Gomes, Scott Tanghe, Julio Gallego-Delgado, Luciana Conde, Leonardo Freire-de-Lima, Ana Carolina Lima, Célio Geraldo Freire-de-Lima, Josué da Costa Lima Junior, Otacílio Moreira, Paulo Totino, Ana Rodriguez, Adriane Regina Todeschini, Alexandre Morrot

**Affiliations:** ^1^Centro de Pesquisas em Tuberculose, Instituto de Microbiologia, Faculdade de Medicina, Universidade Federal do Rio de Janeiro, Rio de Janeiro, Brazil; ^2^Division of Parasitology, Department of Microbiology, New York University School of Medicine, New York City, NY, United States; ^3^Instituto de Biofísica Carlos Chagas Filho IBCCF, Universidade Federal do Rio de Janeiro, Rio de Janeiro, Brazil; ^4^Instituto Oswaldo Cruz, Fiocruz, Rio de Janeiro, Brazil; ^5^Laboratório de Glicobiologia Estrutural e Funcional, Instituto de Biofísica Carlos Chagas Filho IBCCF, Universidade Federal do Rio de Janeiro, Rio de Janeiro, Brazil; ^6^Laboratório de Imunoparasitologia, Instituto Oswaldo Cruz, Fiocruz, Rio de Janeiro, Brazil

**Keywords:** *Plasmodium falciparum*, glycobyology, cerebral malaria, treatment strategies, parasites

## Abstract

Cerebral malaria (CM) is a clinical syndrome involving irreversible and lethal signs of brain injury associated to infection by parasites of the genus *Plasmodium*. The pathogenesis of CM derives from infection-induced proinflammatory cytokines associated with cytoadherence of parasitized red blood cells to brain microvasculature. Glycoconjugates are very abundant in the surface of *Plasmodium* spp., and are critical mediators of parasite virulence in host–pathogen interactions. Herein, we show that 6-Diazo-5-oxo-L-norleucine (DON) therapeutically used for blocking hexosamine biosynthetic pathway leads to recovery in experimental murine cerebral malaria. DON-induced protection was associated with decreased parasitism, which severely reduced *Plasmodium* transmission to mosquitoes. These findings point to a potential use of DON in combination therapies against malaria.

## Introduction

Malaria is a parasitic disease caused by the protozoan *Plasmodium*. The more severe clinical manifestation of the disease affects the microvasculature of the blood–brain barrier resulting in the cerebral form of disease (Polimeni and Prato, 2014). Cerebral malaria is associated with infection by *Plasmodium falciparum*, which affects over half a million individuals annually (World Health Organization, 2015), most of them being represented by children in sub-Saharan Africa (Idro et al., 2010). The use of animal models of infection has allowed us to accumulate knowledge about the pathophysiology of disease. Experimental murine models using C57BL/6 mice infected with *Plasmodium berghei* ANKA (PbA) are able to reproduce the clinical signs of human cerebral malaria (de Oca et al., 2013). Different studies have consistently shown the requirement for T lymphocytes in the pathogenesis of cerebral malaria (Renia et al., 2006) by demonstrating that infection in mice deficient in beta-2-microglobulin gene as well as mice lacking functional CD8^+^ T cells are resistant to experimental cerebral malaria (Belnoue et al., 2002).

Additional studies have shown that the trafficking of CD8^+^ T lymphocytes to the brain is a critical factor involved in the pathogenesis of experimental malaria after *Pb*A infection (Howland et al., 2015a). Antigen-specific CD8^+^ T cells are able to recognize parasite antigens cross-presented via MHC class I by brain endothelium that is subject to cellular interaction with infected red blood cells (Howland et al., 2015b). The triggering of perforin-mediated endothelial cell dysfunction by activated effector CD8^+^ T cells is a determinant for disease pathogenesis (Jordan and Hunter, 2010). *In vivo* administration of 6-diazo-5-oxo-L-norleucine (DON) late in the course of infection, when the mice have already triggered endothelial dysfunction due to the action of CD8^+^ T cell responses, was able to rescue the clinical manifestation signs of cerebral malaria. These studies have suggested that DON treatment has a protective effect by decreasing function of activated effector CD8^+^ T cells (Gordon et al., 2015).

The inhibitory effect of DON in blocking the pathogenic role of CD8^+^T cells may account to its role in the rescue of mice with experimental cerebral malaria (Howland et al., 2015a). DON is a glutamine antagonist used as inhibitor of different glutamine-utilizing enzymes such as glutaminase, aminotransferases, and glutamine synthetase (Pinkus, 1977). Among those enzymes is the glutamine-fructose-6-phosphate transaminase (GFPT) (Ginsburg, 2006), the rate-limiting enzyme of the hexosamine biosynthetic pathway which transfers amino group from glutamine to the fructose-6-phosphate to form glucosamine-6-phosphate (GlcN6P). GFPT participates in the synthesis of uridine diphosphate *N*-acetylglucosamine (UDP-GlcNAc), used for biosynthesis of glycoproteins, glycosaminoglycans, proteoglycans, and glycolipids (Vasconcelos-Dos-Santos et al., 2015).

Hexosamine biosynthetic pathway is predicted to be essential in *P. falciparum* as it feeds the biosynthesis of glycosylphosphatidylinositol (GPI) anchors which are required for parasite survival and infectivity, thus contributing to malaria pathogenesis (Krishnegowda et al., 2005). Furthermore, GlcNAc can also be incorporated to short N-glycans composed of one or two residues of the sugar (Bushkin et al., 2010; Macedo et al., 2010; Samuelson and Robbins, 2015). N-linked glycosylation is essential for the parasite given that the N-glycosylation blocker tunicamycin arrested parasite development (Dieckmann-Schuppert et al., 1992). Those inhibitory effects of DON on the hexosamine biosynthetic pathway account for its antiparasitic activity of both *in vitro* and *in vivo* (Queen et al., 1990; Waknine-Grinberg et al., 2010). In the present study we investigated the importance of GFPT as a potential malarial transmission-blocking target required for successful development cycle of *P. berghei* ANKA within the vertebrate host.

## Materials and Methods

### Ethics Statement

Protocols for animal experimentation ere used in accordance with the guidelines for the animal welfare regulations set by the National Institutes of Health, United States. The study was approved by the Research Ethics Committee of Federal University of Rio de Janeiro (Protocol No. IMPPG040-07/16). Protocols for animal were approved by the Institutional Ethical Committees in accordance with international guidelines. All animal experimentation was performed in accordance with the terms of the Brazilian guidelines for animal welfare regulations.

### Animals and Infection

C57Bl/6 mice and Swiss Webster (6–8 week-old females) were obtained from The Jackson Laboratories. C57Bl/6 mice were intraperitoneally infected (*i.p.*) with *P. berghei* ANKA expressing GFP (*P. berghei* ANKA) by injection of 1 × 10^6^ *Pb*A-infected red blood cells (*Pb*ARBC) in cerebral malaria experiments. This infection protocol was used for Swiss Webster mice were in transmission-blocking experiments upon infection with *Plasmodium yoelli* -luciferase. The parasitemia was determined by GIEMSA staining of peripheral blood smears during the course of infection. Infected mice were monitored for progression of CM signs using a 5-point clinical scoring system that rates mice from a score of 0 (no signs) to 5 (moribund) based on their appearance and behavior, as previously described (Plaimas et al., 2013). Mice weighing ∼20 g were intraperitoneally injected with DON (1.3 mg/kg), and/or GlcN (40 mg/kg) in 100 μL PBS, beginning the treatment at the first clinical symptoms of CM until day 11. In transmission-blocking experiments, Swiss Webster mice infected with 10^3^ of luciferase-expressing *P. yoelli* were daily treated with doses of DON (0.5 mg/kg) or vehicle (saline) intraperitoneally administered, starting on day 4 after the infection.

### Bioimaging Detection of Asexual Blood-Stage Parasites *in vivo*

Groups of five mice were infected with 10^3^ blood-stage *P. yoelli*-luciferase by intraperitoneal injection. On day 7 post-infection, mice were anesthetized by isoflurane inhalation and injected with 150 mg/kg D-luciferin potassium salt substrate for bioimaging using IVIS imager (Lumina II *in vivo* Imaging System; Perkin-Elmer). Light intensity was measured in each mouse to determine the baseline of infection levels before treatment. Mice were then treated with DON administered daily by intraperitonial injection (0.5 mg/kg) and saline as vehicle (from days 4 to 7 post-infection). A group of negative-control mice were treated with vehicle alone. On day 7 post-infection, mice were imaged to determine the parasite burden.

### Transmission-Blocking of *P. yoelli-*Luciferase to Mosquitoes

Groups of five Swiss Webster mice were infected with 10^3^ *P. yoelli*-luciferase and both gametocytemia and parasitemia were detected at 4 days post-infection by blood smear (five mice per group in three independent experiments). Mice were then alternatively treated with DON administered daily by intraperitoneal injection (0.5 mg/kg). The vehicle control group was obtained by injecting saline only. At day 4 post-infection, mice were anesthetized with ketamine (3,500 mg/kg), xylazine (300 mg/kg) and groups of 100 *Anopheles stephensi* mosquitoes were allowed to feed on each group for 20 min with feeding disruption once every 5 min. The infected mosquitoes were then incubated at 18°C for 11 days to allow oocyte formation. Afterward, the mosquitoes were dissected and their midguts removed, and homogenized for parasite detection after incubation with D-Luciferin potassium salt (200 μg/ml). Luminescence was measured by using a microplateplate reader (PerkinElmer).

### RNA Extraction and cDNA Synthesis

Total RNA was extracted from 1 mL blood using RNeasy^®^mini kit (QIAGEN). Samples were collected from infected and uninfected animals, and immediately mixed with 350 μL of lysis buffer (RLT buffer). RNA was extracted according to manufacturer’s protocol and resuspended in 30 μL of elution buffer. RNA was stored at -80°C until use. RNA samples were treated with DNAse I (Sigma-Aldrich), and the quantification was performed using a ND2000 Nanodrop (Thermo Fisher Scientific). The reverse transcription was performed using the Superscript IV ViloMastermix (Invitrogen), following manufacture’s instructions.

### Quantification of PbCCp3 and PbHSP70 Transcripts by RT-qPCR

The comparative quantification of *P. berghei* gametocyte forms between treated (1,3 mg/kg/day of DON) versus control mice was performed by PbCCp3 mRNA detection in blood samples. In parallel, the PbHSP70 mRNA levels were also quantified to detect all the parasite evolutive forms (Lavazec et al., 2009). For real-time PCR assays, 2 μl cDNA were used in a final reaction volume of 20 μL, with 10 μL of Power SYBR Green PCR Master Mix 2X (Life Technologies, Foster City, CA, United States), 300 nM of each PbCCp3 (sense 5′-CTGCAGCTATTTATGATGGT, antisense 5′-TCATCACTTTCATCACCTTT) or PHSP70 (sense 51-AGAGAAGCAGCTGAAACAGC,antisense 5′-TCCCTTTAATAAATCATGGC) primers. PCR cycling conditions were used with first step at 95°C for 10 min, followed by 40 cycles at 95°C for 15 s and 60°C for 1 min. To check the primers specificity, melting curves were generated after the 40 cycles. To obtain the Threshold Cycle (Ct), threshold was set at 0.02. The comparative quantification was expressed as 2^-ΔCt^, where ΔCt = Ct_Treatedmouse_ – Ct_Untreatedmouse_. RT-qPCR assays were performed in a ViiA7 Real Time PCR System (Applied Biosystems), in technical duplicates and biological triplicates.

### *In vitro* Growth of Asexual Erythrocytic Forms of *Plasmodium falciparum*

The erythrocytic asexual stage of *P. falciparum* (3D7 strain) was cultured in 96 well plates for 96 h, in RPMI media containing 25 mM HEPES, 25 mM sodium bicarbonate, 10 μg/ml gentamycin, 0.5 mM hypoxanthine, at pH 6.75 in atmospheric conditions (5% CO_2_, 5% O_2_, and 90% N_2_). Alternative cultures were done in the presence of DON (50 μM). To set up the culture the parasites were synchronized using MACS cell separation column (MiltenyiBiotec) and the parasitemia was maintained below 5%. The parasite load was determined by GIEMSA staining of peripheral blood smear.

### Mitochondrial Transmembrane Potential Assay

The effect of DON on parasite viability as well as development in *P. falciparum* cultures was examined by flow cytometry using rhodamine 123 (Invitrogen) as indicator of mitochondrial activity, as previously described (Totino et al., 2008). Briefly, cultures were washed in RPMI medium (RPMI-1640, 25 mM Hepes, 0.2% glucose, 23 mM sodium bicarbonate) and then incubated at 37°C for 5 min with 1 μg/ml rhodamine in RPMI. Rhodamine solution was removed by centrifugation and cultures were washed once before incubation for 30 min in complete medium alone. Afterward, cultures were ressuspended in fresh complete medium and analyzed in a flow cytometer (FACSVerse, Becton Dickinson) using 488-nm blue laser and 527/32 bandpass filter. Non-parasitized RBC samples stained with rodamine were used as negative control in flow cytometry analysis.

### Statistical Analysis

Statistical analyses were performed with GraphPad Prism 5 software. Statistical differences between mean values were evaluated by non-parametric Student’s *t*-test or analyzed with ANOVA and Bonferroni *post hoc* test. Results were expressed as mean ± standard deviation (SD), and differences between control and treated group were considered statistically significant when *p* ≤ 0.05.

## Results

### Requirement of Glutamine-Fructose-6-Phosphate Transaminase for *in vitro* Growth of Erythrocytic Forms of *Plasmodium* Parasites

**FIGURE 1:**
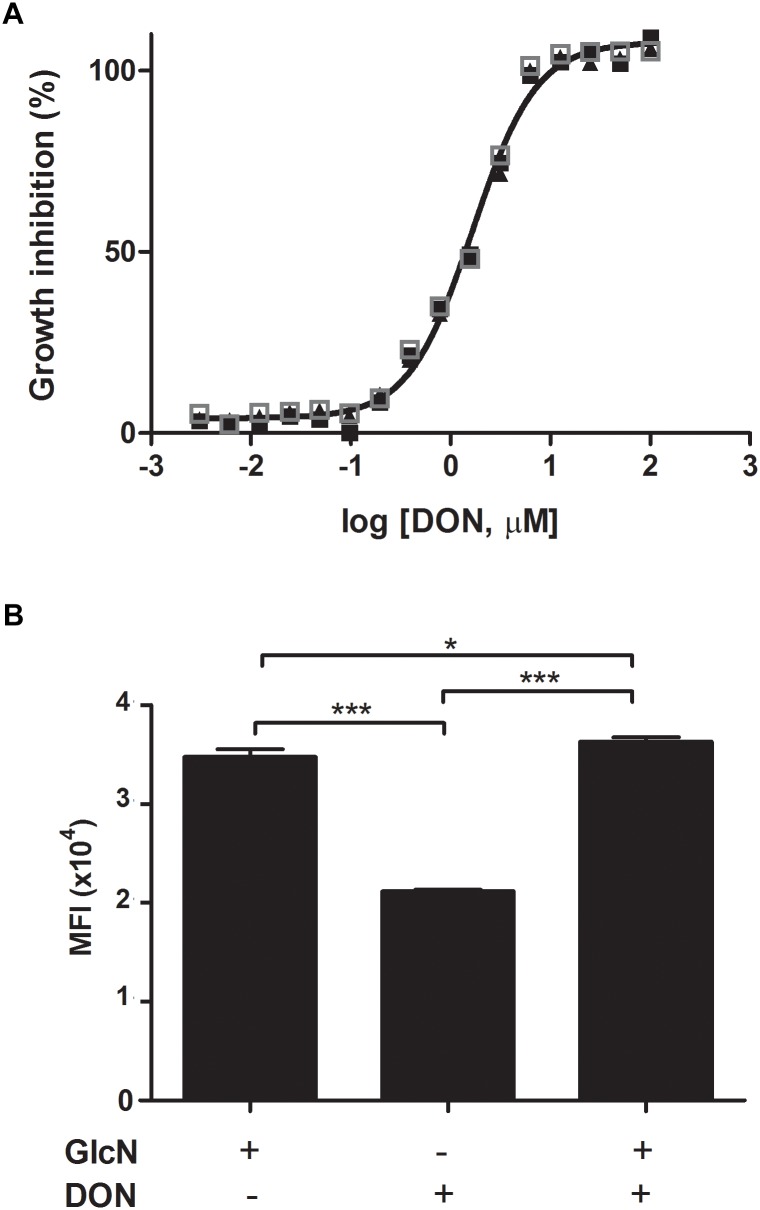
The hexosamine biosynthetic pathway is required for the growth of erythrocytic forms of *Plasmodium* parasites. **(A)** *Plasmodium falciparum* 3D7 strain parasites were incubated with fresh human erythrocytes in the absence or presence of increased doses of DON. Parasitemia was determined 4 days later. The IC_50_ value of DON (1.6 μM) was determined as the concentration that inhibits parasite growth by 50%. The concentration-dependent inhibitory dose-curve data was plotted as percentage inhibition normalized to normal infected-controls in the absence of inhibitor with applied curve fits calculated using GraphPad Prism. **(B)** The inhibitory effect of DON on *P. falciparum* 3D7 strain parasites incubated with erythrocytes was reverted by the addition of exogenous GlcN (125 μM) to the cultures and parasitemia was determined at day 4 pi. The results indicate a requirement of the hexosamine biosynthetic pathway for the parasite blood-stage growth. Data are means ± SD and represent the results of three independent experiments. Differences between groups are significant (^∗^*p* < 0.05, ^∗∗∗^*p* < 0.001).

We first analyzed the rates of parasite growth in cultures of *P. falciparum*-infected erythrocytes in the presence or absence of DON. Analysis of *in vitro* cultures of *P. falciparum* in erythrocyte cells showed a significant lower growth rate of the parasites in the presence of DON (IC50 = 1.6 μM) as compared to controls of erythrocytes supplemented with medium only (Figure 1A). Since DON is not a specific inhibitor of GFPT, as it inhibits other amidotransferases, we tested whether the addition of glucosamine (GlcN) could restore parasite growth by bypassing GFPT inhibition by DON. GlcN is easily transported by the glucose transporter systems, and then phosphorylated by hexokinase to glucosamine 6-phosphate. Glucosamine 6-phosphate directly enters the hexosamine biosynthetic pathway bypassing the key regulatory enzyme GFPT, resulting in an increase in UDP-GlcNAc. Indeed, the inhibition of GFPT by DON can be compensated by the exogenous addition of GlcN (Figure 1B). Our findings indicate that the inhibitory effect on the parasite growth in red blood cells cultured in presence of DON was reverted upon exogenous addition of glucosamine in infected erythrocytes, therefore indicating a requirement for the UDP-GlcNA chexosamine downstream product of GFPT in the parasite blood-stage cycle.

**FIGURE 2:**
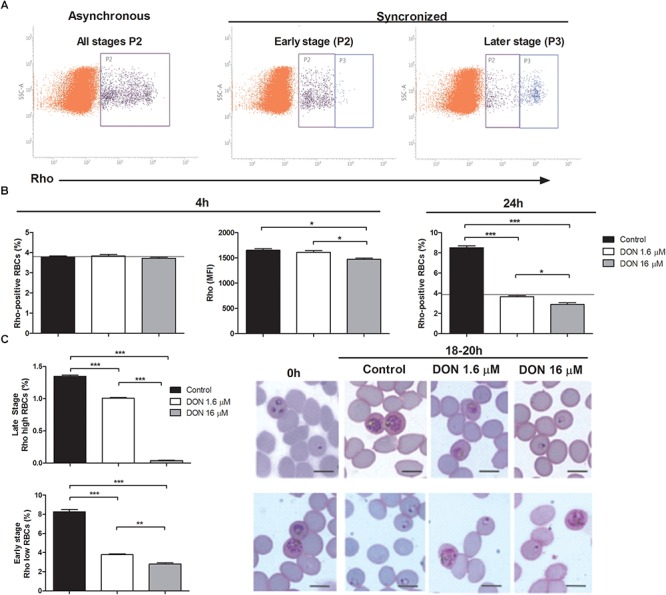
Effect of DON on parasite viability and stage-specific development measured by mitochondrial activity using rhodamine 123 (Rho) staining. **(A)** Representative flow cytometry analysis of mitochondrial activity in asynchronous (P2: Rho-positive RBCs) and synchronized *P. falciparum* culture enriched with early (P2: Rho low RBCs) or late (P3: Rho high RBCs) stages. **(B)** Asynchronous culture was incubated in absence (Control) or presence of DON (1.6 and 16 μM) and, then, parasite viability was evaluated after 4 h by measuring both percentage of Rho-positive RBCs (P2 in A, asynchronous) and Rho fluorescence intensity (MFI) in this RBCs population. Parasite growth was also estimated by determining percentage of Rho-positive RBCs after 24 h. The horizontal black lines represent initial parasitemia. **(C)** Stage-specific effect of DON was tested using synchronized cultures (0 h) in early (upper panel) or late (lower panel) stages, as shown in (**A**, synchronized). Cultures were incubated in absence (Control) or presence of DON (1.6 and 16 μM) and parasite development was evaluated after 18–20 h by determining the percentage of late (Rho high RBCs, upper panel) and early stages (Rho low RBCs; lower panel) in flow cytometry analysis. In addition, parasite morphology was examined by Giemsa-stained smears using light microscopy. Data are means ± SD and differences between groups are significant (^∗^*p* < 0.05, ^∗∗^*p* < 0.01, ^∗∗∗^*p* < 0.001). Scale bars in **(C)** indicate 7 μm.

To better understand the antimalarial effect of DON on blood-stage forms of *Plasmodium* parasites, we performed mitochondrial activity assay by flow cytometry using rhodamine 123 (Rho) to evaluate parasite viability as well as stage-specific parasite development (Figure 2A). Rho is incorporated by active mitochondria of living parasites in a proportional manner of the developmental stage, differentially staining early (Rho low RBCs) and late (Rho high RBCs) stages of intraerythrocytic development (Figure 2A, synchronized). Using this approach, we observed no effect of 1.6 μM DON on parasite viability in asynchronous *P. falciparum* culture treated for 4 h, as the number of Rho-positive RBCs and Rho fluorescence intensity (MFI) were similarly detected in control cultures, while 16 μM DON induced a slight, although statistically significant, decrease of Rho MFI at 4 h (Figure 2B). In contrast, both concentrations of DON (1.6 and 16 μM) notably inhibited the increase of Rho-positive RBC levels after 24 h of culture (Figure 2B), indicating that rather than an immediate cytotoxic effect on parasite viability, DON acts arresting plasmodial development.

Indeed, when we used early-stage synchronized culture to study parasite development under DON pressure, a decreased number of late stage parasites (Rho high RBCs) was detected in DON-treated cultures, with a remarkable effect of 16 μM DON (Figure 2C, upper panel). Parallel examination of such cultures by light microscopy showed that parasite development was arrested at ring and late trophozoite stages at the 16 and 1.6 μM concentration, respectively. Moreover, a significant reduction of newly invaded early forms was also observed after incubating late-stage cultures in the presence of DON, which induced appearance of late-stage parasites displaying abnormal morphology, as evidenced by microscopy analysis (Figure 2C, lower panel). These results indicates that DON acts arresting stage-specific development of *Plasmodium* parasites in a dose-dependent manner, while invariably affects late-stage maturation and, consequently, the increase of parasitemia.

**FIGURE 3:**
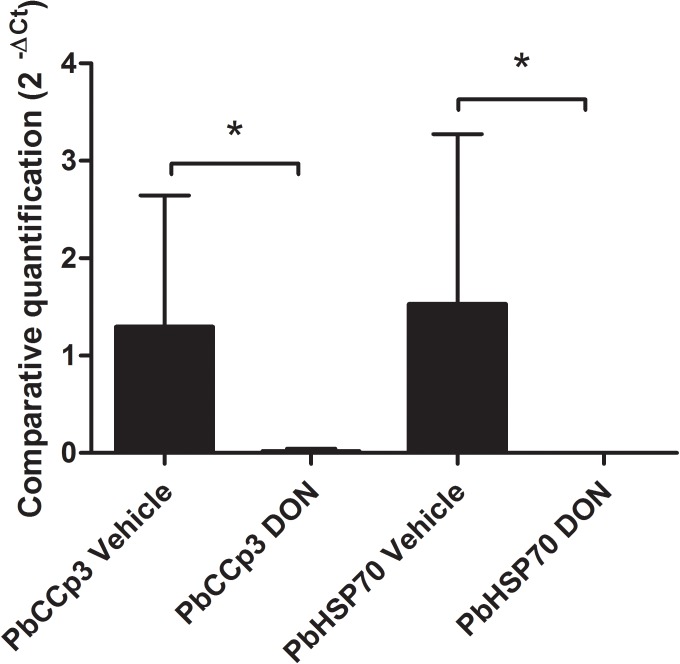
Inhibitory effect of DON on malaria erythrocytic stage development. CB57BL/6 mice were injected intraperitoneally with DON (1.3 mg/kg), administered consecutively every day, during the course of infection with 10^3^ blood-stage *Plasmodium berghei* ANKA parasites. Gene expression levels by RT-qPCR at day 6 post-infection allow the detection of gametocytes and asexual erythrocytic forms of *P. berghei*. It was possible to verify the presence of circulating both forms in the whole blood of the animals used in the study and confirm the anti-parasitic action of the drug. Statistical analysis was performed using ΔCt values and we observed that treated animals showed significantly higher parasite density than untreated animals as regards to asexual and sexual forms of *P. berghei*. Data are means ± SD and differences between groups are significant (^∗^*p* < 0.05).

To access the inhibitory effect of DON on malaria erythrocytic stage development in the experimental murine model of infection, CB57BL/6 mice treated with DON (1.3 mg/kg) was injected intraperitoneally, administered consecutively every day, during the course of infection with 10^3^ blood-stage *P. berghei* ANKA parasites. RT-qPCR analysis by targeting PbHSP70 mRNA, which is constitutively expressed in all the *P. berghei* evolutive forms (Figure 3), confirmed a reduction in the blood-stage parasitemia at day 6 post-infection. In addition, the detection of *P. berghei* gametocyte forms, by accessing the PbCCp3 mRNA levels, indicated significantly lower levels of transcripts (59,96 times) from DON-treated group as compared to control (vehicle) group (Figure 3).

### *In vivo* Inhibition of GFPT in the Course of Experimental Malaria Prevents *Plasmodium* Development, Transmission, and Disease Pathology

**FIGURE 4:**
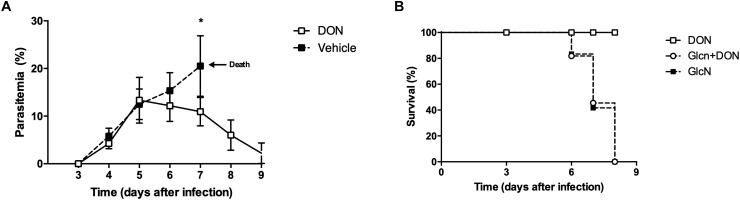
Inhibition of GFPT in the onset of malaria blood-stage infection attenuates the parasitemia and establishment of disease pathology. C57BL/6 mice were infected with 10^3^ blood-stage *P. berghei* ANKA by intraperitoneal injection. DON (1.3 mg/kg) and/or GlcN (40 mg/kg) in 200 μL saline were *i.p*. daily after the day 5 *pi*. The control group was injected with saline. Results from **(A)** peripheral blood parasitemia **(B)** and Kaplan–Meier survival plots were obtained from three independent experiments. The results shown are combined from three independent experiments performed with five mice per group. Data are means ± SD and differences between groups are significant (^∗^*p* < 0.05).

Our results indicating an inhibitory effect of DON on blood-stage *P. falciparum* allowed us to investigate a possible effect of this inhibitor on the infection *in vivo*. For this purpose, we first used an experimental model of murine cerebral malaria established by *P. berghei* ANKA infection in C57BL/6 mice which reproduces the critical pathological features of human cerebral malaria. Recent studies have shown that the therapeutic administration of DON in the late phase of murine cerebral malaria, from day 6 post-infection of *P. berghei* ANKA infection in CB57BL/6 mice, has an inhibitory effect on parasitemia, further showing that the glutamine analog is able to prevent pathogenic CD8^+^ T cell responses (Gordon et al., 2015). In the current study, our results demonstrate that when DON (1.3 mg/kg) was intraperitoneally injected beginning at day 6 post-infection and administered consecutively every day, during 4 days, there was a significant reduction of parasitemia in comparison to controls treated with saline alone (Figure 4A). All infected control animals developed neurological signs associated with murine cerebral malaria, showing a 100% mortality around day 7 post-infection. However, the animals treated with DON displayed a survival rate of 100% at 9 days post-infection (Figure 4B). It is worth of note that mice treated with DON and GlcN present a survival curve similar to the control (untreated mice), in which 80% died up to day 7 post-infection, and all died by day 8 post-infection (Figure 4B). These results clearly showed that GlcN reverts DON effect on mice survival, reinforcing the role of hexosamine biosynthetic pathway in parasite infection.

**FIGURE 5:**
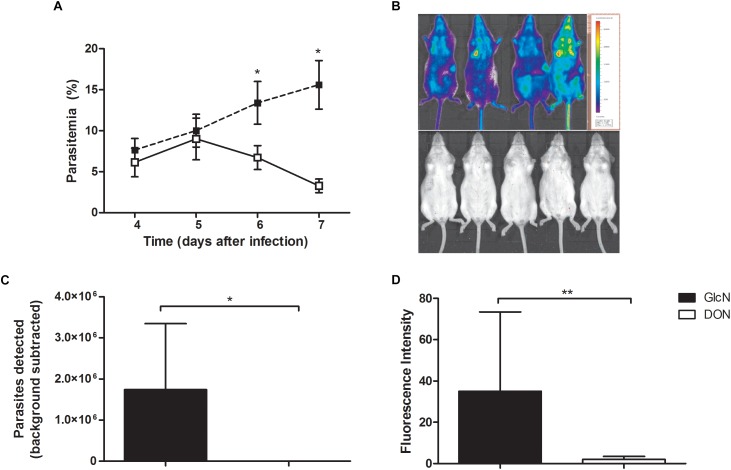
Potential effect of DON as malarial transmission-blocking agent preventing *Plasmodium* developmental cycle. Swiss Webster mice infected with 5 × 10^-5^ with luciferase-expressing *P. berghei* ANKA were treated with daily doses of DON (0.5 mg/kg) or vehicle (saline) as control from days 4 to 7 post-infection, administered intraperitoneally twice per day. Afterward, mice were injected (*i.p.*) with 150 mg/kg body weight of D-luciferin potassium salt substrate for bioimaging detection of asexual blood-stage parasites *in vivo* using IVIS imager (Lumina II In Vivo Imaging System; Perkin-Elmer). **(A)** Peripheral blood parasitemia were obtained from three independent experiments. The results are shown as means ± SE combined from three independent experiments performed with five mice per group. **(B)** Rainbow scale analysis of luciferase-tagged *P. berghei* ANKA parasite burden as representative bioluminescent images. **(C)** Quantification of the total luminescence intensity from whole body imaging of mice (*n* = 5 mice per group). **(D)** Transmission-blocking effect of DON in the sexual phase of the parasite life cycle. Infected Swiss Webster mice were alternatively treated with daily doses of DON (1.3 mg/kg) or vehicle as control from the beginning of infection (day 0) and 4 days later, experimental mouse groups (*n* = 5) were used to assay for the oocysts formation in the vector. Female *Anopheles stephensi* mosquitos (100 per experimental mouse group) were allowed to feed for 20 min and after 11 days later their maintenance at 18°C, the oocyst formation was determined from macerated midguts (*n* = 20 midguts per group) with addition of D-Luciferin potassium salt (200 μg/ml) for fluorescence intensity detection. Data are means ± SD and differences between groups are significant (^∗^*p* < 0.05, ^∗∗^*p* < 0.01).

We then investigated the ability of DON to act as a potential malarial transmission-blocking agent in view of its inhibitory effect on *Plasmodium* blood-stage development. For this purpose, we used the outbred Swiss Webster mouse model of cerebral malaria, as these are commonly used for testing vaccines and drugs that are expected to be effective in genetically heterogeneous background populations (Martins et al., 2009). Infection of these mice with *P. berghei* ANKA rapidly increased parasitemia but did not induce signs of cerebral malaria allowing the study of mouse-mosquito transmission. Corroborating our findings with CB57BL/6 mice, we detected a significant reduction in parasitemia indexes in infected Swiss mice treated with DON as compared to controls groups (Figure 5A). Analysis by chemiluminescence detection of luciferase-tagged *P. berghei* ANKA parasites *in vivo* (Figure 5B,C) confirmed a reduction in the blood-stage parasitemia.

We next investigated the potential effect of DON on *Plasmodium* transmission from vertebrate to mosquito. Swiss Webster mice were infected with 5 × 10^-5^ luciferase-expressing *P. berghei* ANKA and either treated or not with daily doses of DON (1.3 mg/kg) administered intraperitoneally from the beginning of infection (day 0). At day 4 post-infection, mice were anesthetized and *Anopheles stephensi* mosquitoes were allowed to feed on each mice group. Following incubation for 11 days, the levels of luciferase-expressing parasites in mosquitos’ midguts were estimated using D-Luciferin potassium salt. Our results demonstrated that, in fact, DON was able to significantly reduce the intensity of parasite transmission compared to control animals not treated with the inhibitor (Figure 5D), thus also indicating an effect of this inhibitor on development of sexual blood stages of malaria parasites.

## Discussion

Malaria is an infectious disease transmitted by mosquitoes and caused by parasitic protozoa of the genus *Plasmodium* (Cox, 2010). More severe cases of the disease may involve neurological complications and encephalopathies characteristic of cerebral malaria (Trampuz et al., 2003). These clinical manifestations are a consequence of the pathological activation of responses mostly triggered by CD8^+^ T lymphocytes directed against the parasite at the brain-endothelial interfaces (Howland et al., 2015a). Malaria prevention methods include mosquito eradication and use of medications able to target the parasite virulence (Ramirez et al., 2009). The prevalence of malaria in endemic area presupposes the combination of several factors, including high human population density, high population density of *Anopheles* mosquitoes, and a high rate of transmission between humans and mosquitoes (Martens and Hall, 2000). When any of these factors is reduced significantly, the parasite is expected to eventually disappear from the endemic regions as it occurred in North America, Europe and regions of the Middle East (Shiff, 2002).

There are a number of drugs available for malaria prevention in endemic areas. Many of these drugs are also used in the treatment of the disease (White, 2004). In cases where the parasite is still sensitive, chloroquine can be used. However, several species of *Plasmodium* are resistant to one or more drugs, so it is often necessary to resort to other drugs or combinations of drugs (White, 1999). Among these are mefloquine, doxycycline or the combination of atovaquone and proguanil (Gomes and Morrot, 2016). Ideally, drugs would be designed to act not only on prevention by acting directly on the parasite growth but also acting on the clinical symptoms by reducing disease severity. In this regard, important considerations are given to targets of carbohydrate metabolism and glycoconjugate biosynthesis pathways. For instance, GPI anchors are abundantly expressed in the surface of *Plasmodium* spp. and are determinants in the induction of parasite virulence in host–pathogen interactions and pathological responses to infection (Gazzinelli et al., 2014), while N-linked glycosylation seems to be essential to parasite grown (Dieckmann-Schuppert et al., 1992).

Previous work showed that glutamine analog DON reduced parasite growth in cultures of *P. falciparum-*infected erythrocytes (Waknine-Grinberg et al., 2010). Recently it was also shown that the activation of pathogenic CD8^+^ T cell responses in the course of cerebral malaria are dependent on glutamine metabolism and *in vivo* administration of DON in the late-phase of experimental cerebral malaria was able to inhibit the pathogenic CD8^+^ T cells responses thus rescuing the clinical manifestation signs of disease (Gordon et al., 2015). In the present study, we show that when administered to susceptible mouse models early in the onset of malaria blood-stage parasite infection, DON significantly decreased the levels of parasitemia thus reducing *P. berghei* ANKA lethality in CB57BL/6 mice. We demonstrated, for the first time, that the effect of DON in experimental malaria is due to inhibition of the aminotransferase activity of rate-limiting enzyme of the hexosamine biosynthetic pathway, GFPT.

Our findings demonstrated a requirement for GFPT activity for growth of erythrocytic forms of *Plasmodium* parasites. *In vitro* culture of *P. falciparum* in erythrocyte cells showed a significant inhibition of parasite growth in the presence of DON, which arrested parasite development at early (16 μM) and late trophozoite (1.6 μM) stages as well as induced abnormal morphology in mature forms. The inhibitory effect of DON was reverted upon exogenous addition of GlcN in infected erythrocytes, thus indicating a requirement for the UDP-GlcNAc downstream product of the hexosamine biosynthetic pathway in the parasite blood-stage cycle. In this line of evidence, coadministration of GlcN to DON treated mice reverts DON effect on mice survival, restoring high parasitemia and mortality of untreated mice. Moreover, our results indicate that the sensitivity of blood-stage forms of *Plasmodium* parasites to DON and GlcN, which reflects the importance of the parasite hexosamine biosynthetic pathway, is not restricted to dividing asexual forms, since our findings indicated that mice treated with DON presented significantly lower parasitemias of both asexual and gametocyte forms when compared with non-treated animals. In this regard, it has been shown that in erythrocytes infected with *P. falciparum* there is an increase of the glutamine influx as compared to normal cells that may reflect a possible increment of the hexosamine biosynthetic pathway needed for parasite blood-stage growth and cycle differentiation (Srivastava et al., 2016). Besides, studies have shown that gametocytes and gametes rely mainly on glycolysis but also display dependence on glutamine metabolism (Srivastava et al., 2016).

Hexosamine biosynthetic pathway is controlled by the rate-limiting enzyme GFPT which belongs to the aminotransferase family (Ginsburg, 2006) and generates UDP-GlcNAc, by *de novo* pathway. UDP-GlcNAc can be also obtained by the salvage pathway, perhaps due to the action of hexokinase that catalyzes the phosphorylation of glucosamine (GlcN) to GlcN-6-P, which then enters the same route as the *de novo* pathway (Sanz et al., 2013). UDP-GlcNAc is a donor substrate used in the production of short N-linked glycans and GPI anchors, both are essentials for parasite survival (Krishnegowda et al., 2005). Inhibition of N-linked glycosylation by tunicamycin, which catalyzes the transfer of GlcNAc-1P from UDP-GlcNAc to dolichol phosphate in the first step of N-linked glycoprotein synthesis (Vasconcelos-Dos-Santos et al., 2015), is lethal for the parasite (Naik et al., 2001). Inhibition of *P. falciparum* GPI by Glucosamine (GlcN) constrains the growth of the parasite in a dose-dependent manner. GlcN specifically arrested the maturation of trophozoites, a stage at which the parasite synthesizes all of its GPI anchor pool, and had no effect during the parasite growth from rings to early trophozoites and from late trophozoites to schizonts and merozoites (Naik et al., 2003).

Besides, addition of GlcNAc to *P. falciparum* culture is one of the most used method to obtain gametocytes stages *in vitro* (Ponnudurai et al., 1986; Fivelman et al., 2007). Our findings demonstrating the blood-stage parasite dependence of the GFPT enzyme may indicate the use of the glutamine metabolism through the hexosamine biosynthetic pathway. The findings indicating that DON is able to act directly on the growth and differentiation of the blood forms of the *Plasmodium* parasites as well as attenuate the pathology of the host inflammatory responses emphasizing its importance in the design of new therapies against malaria. Drugs able to reduce the parasite blood-stage cycle, affecting the sexual stages of *Plasmodium* parasites should have an immediate impact on mosquito infection (oocyst prevalence and intensity) and the transmission intensity of vectors in the endemic areas of malaria (Dyer and Day, 2000). In this regards, the therapeutic use of analogs of DON should provide a potential target for its use as transmission-blocking drugs against malaria.

## Author Contributions

PG, AL, OM, and PT conducted the experiments. PG, AL, OM, and PT acquired the data. PG, LC, AL, and OM analyzed the data. PG, ST, JG-D, JLJ, PT, AR, AT, and AM designed the research studies. LF-d-L, CF-d-L, OM, PT, AR, AT, and AM provided the reagents. PG, JLJ, OM, PT, and AM wrote the manuscript. All the authors have read and approved the final manuscript.

## Conflict of Interest Statement

The authors declare that the research was conducted in the absence of any commercial or financial relationships that could be construed as a potential conflict of interest.
